# Stress Corrosion Cracking Behavior of TP 439 and 690 TT under Film-Forming Amine Environment

**DOI:** 10.1155/2021/6668537

**Published:** 2021-06-08

**Authors:** Lu Jundong, Jiang Xiaobin, Sun Ke, Liu Bin, Li Xinmin, Ni Qinwen

**Affiliations:** ^1^Suzhou Nuclear Power Research Institute, Suzhou, 215800 Jiangsu, China; ^2^Guangdong Nuclear Power Joint Venture Co., Ltd., Shenzhen, 518000 Guangdong, China

## Abstract

Film-forming amines have been widely used in thermal power plants for maintenance after shutdown, and there are more and more applications and researches in nuclear power secondary circuits for this purpose. However, in the direction of stress corrosion cracking, there is not much research on the influence of film-forming amines on metal materials. This article uses the high temperature slow strain rate test (SSRT) method to evaluate the influence of a commercial film-forming amine on the stress corrosion cracking behavior of two conventional island materials for PWR nuclear power plants. These two metal materials are the heat exchange tube materials of the high-pressure heater and steam generator in the high-temperature operation area of the secondary circuit of a nuclear power plant: TP 439 stainless steel and 690 TT alloy. The test analyzed the mechanical properties and fracture morphology. The test results show that in the test concentration range (<5 mg/kg), the film-forming amine will not affect the SCC of TP 439 stainless steel and 690 TT alloy under the condition of slow strain rate. The behavior has a significant impact. In practical applications, the general dosage of film-forming amine is 1-2 mg/kg. This data is lower than the film-forming amine concentration used in the experiment. Therefore, there is no need to worry about the obvious impact on the SCC behavior of TP 439 stainless steel and 690 TT alloy.

## 1. Introduction

Film-forming amine (FA) is a specific chemical which can form a hydrophobic layer on the metal surface in liquid or vapor phase. This type of chemicals usually contains a primary, secondary, or tertiary amine structure (or a combination thereof) linked to a hydrocarbon chain, usually with more than 10 carbon atoms, such as octadecylamine and oleyl-1,3-propanediamine. Commercial film-forming amine products (FAP) are simply a FA (or multiple), or a compound formed by the dispersion and mixing of a FA (or multiple) and other chemicals.

Presently, in the thermal power industry, the utilization of FAPs into implementing medium- and long-term boiler maintenance after shutdown has become one of the main maintenance methods [1, 2]. The operating experiences of many thermal power plants showed that the shutdown protection of FAPs can effectively reduce the corrosion rate during the shutdown period. The unit has the following advantages after startup: water quality qualified time is shortened and start-up time is shortened.

In the nuclear power industry, there is almost no relevant literature reported. Since 2011, the successful utilization of FAP (ODACON) in Almaraz 1/2# units has attracted the attention of researchers [3, 4]. As a new choice of water chemistry methods to reduce corrosion rate, research institutions that are at the forefront of research in the nuclear power industry have started corresponding research plans.

The published literatures study the physical and chemical properties of FAs [4–7], dissociation constant and distribution coefficient [8, 9], reaction mechanism and influence on metal materials [10–16], the influence on the chemical parameters in the loop such as pH and conductivity [17–20], the decomposition products [18, 21], the influence on the heat exchange performance [22–26], and the influence on the online chemical instrument detection [27]. However, there is not much research work on the stress corrosion cracking behavior of materials at sensitive locations in the FA environment [28].This article is conducting related research work, using a commercial FA that has been applied on the secondary circuit side of a PWR nuclear power plant for evaluation. The test metal material is the heat exchange tube material of the high-pressure heater and steam generator in the high-temperature operation area of the secondary circuit of a nuclear power plant: TP 439 stainless steel and 690 TT alloy. Different from the information [28], the evaluation in this paper is completed by the high temperature slow strain rate test (SSRT) method.

## 2. Experimental

**Figure 1:**
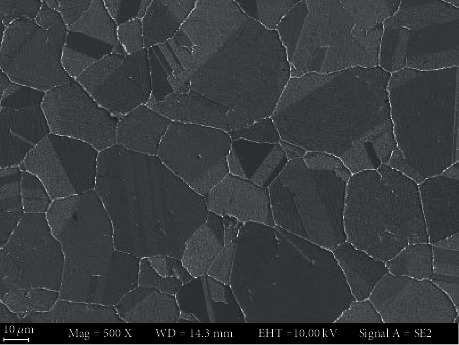
The SEM microstructure of 690 TT alloy. FA: a commercial FA product; emulsion, 1% content.

The materials used in present work is TP 439 stainless steel (SS) and 690 TT alloy. The chemical compositions of TP 439 SS and 690 TT are shown in Table 1.  Figure 1 shows the microstructure morphology of 690 TT alloy. The structure of 690 TT alloy presents a typical equiaxed austenite type, with carbides distributed continuously at the grain boundaries.  Table 2 shows the components of the 690 TT used. The slow strain rate test (SSRT) tests for TP 439 SS and 690 TT were conducted in simulated secondary water environment according to the GB/T 15970.7-2000.

**Figure 2:**
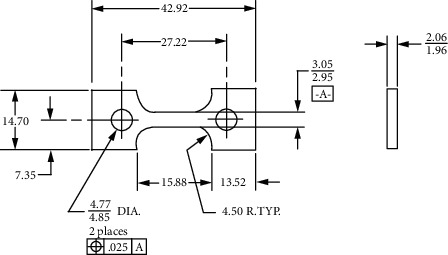
SSRT test specimen size (mm).

The size of the sample is shown in Figure 2. Prior to the test, the samples were cleaned in ethanol using an ultrasonic cleaner, then dried and sealed for later use. The SSRT tests were conducted using the American CORTEST high-temperature and high-pressure slow strain rate tensile tester.

The strain rate for TP 439 SS and 690 TT is 1 × 10^−6^/s and 5 × 10^−7^/s, respectively.

The chemical environment of each test are shown in Table 3.

## 3. Results and Discussion

### 3.1. Analysis of Mechanical Properties

The main principle of macroscopic evaluation of SCC sensitivity is that SCC will cause a significant decrease in material plasticity indicators, such as the material's maximum breaking stress, total elongation, reduction of area, and fracture absorption energy (area under the load-elongation curve). Comparing the same sample exposed to the test environment and exposed to the inert environment, the farther the ratio deviates, the higher the cracking sensitivity.

(1) $$\begin{array}{c} I_{\text{scc}}=\left(1-\frac{t_{0}-t}{t_{0}}\right)\times 100\%. \end{array}$$

In the formula *I*_scc_ represents the stress corrosion cracking sensitivity index of the sample, *t*_0_ is the test result of the sample in an inert medium, and *t* is the test result of the sample in the test environment.

Since the fracture absorption energy of the sample is the area under the load-elongation curve, which contains two parameters of load and elongation, it is relatively comprehensive, so the fracture absorption energy is used to calculate the stress corrosion sensitivity index of the material in different environments. From the calculation results, the stress corrosion sensitivity of TP 439 SS and 690 TT alloy is basically the same in FA and non-FA environments. The use of FAs will not significantly affect the SCC behavior of TP 439 SS and 690 TT alloy under slow strain rate conditions.

**Figure 3:**
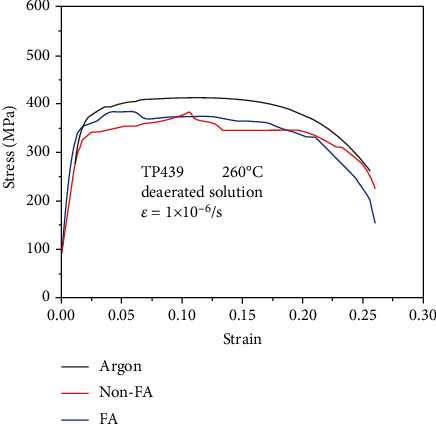
The stress-strain curves of TP 439 SS in different environments.

The stress-strain curve of TP 439 SS in FA, non-FA, and argon atmosphere is plotted in Figure 3. As is seen in the figure, the stress and strain of TP 439 SS in the three environments have all undergone elastic deformation → yield → plastic deformation → reach tensile strength → fracture process. The slow strain rate test data of the sample is listed in Table 3. The stress-strain curves of TP 439 SS in the two environments are basically the same and lower in compared with that in the inert gas argon, so the TP 439 SS has the susceptibility to stress corrosion cracking.

**Figure 4:**
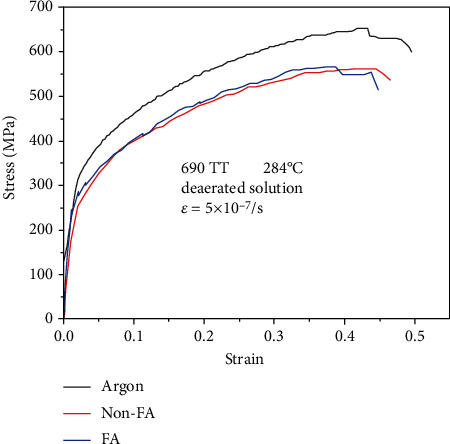
The stress-strain curves of 690 TT alloy in different environments.

The stress-strain curves of 690 TT alloy in FA, non-FA, and argon atmosphere are plotted in Figure 4. As is seen in Figure 3, it is obvious that the stress corrosion sensitivity of 690 TT alloy is basically equal in the FA and non-FA environments. Alloy 690 TT also has the susceptibility to stress corrosion cracking.

### 3.2. Fracture Morphology Analysis

#### 3.2.1. Analysis of the Fracture Surface of TP 439 Stainless Steel

**Figure 5:**
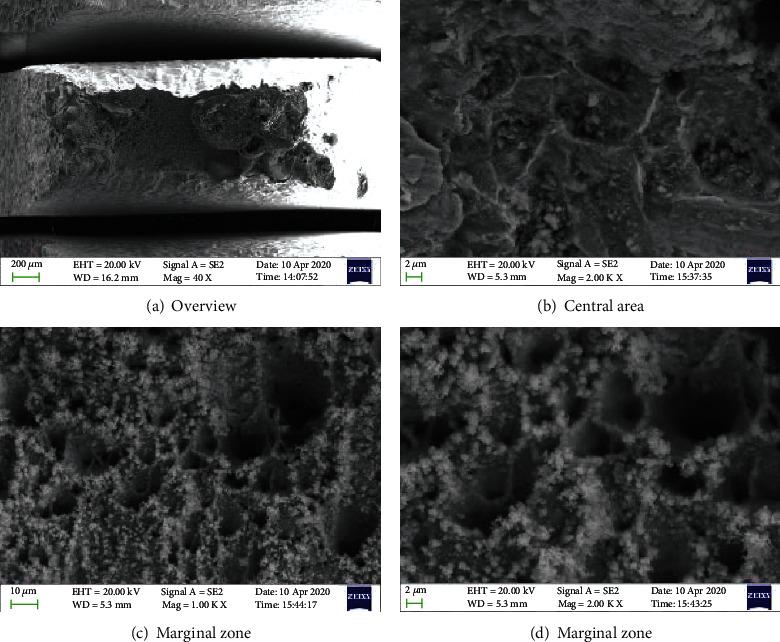
The fracture morphologies for TP 439 SS after SSRT test solution with FA.

**Figure 6:**
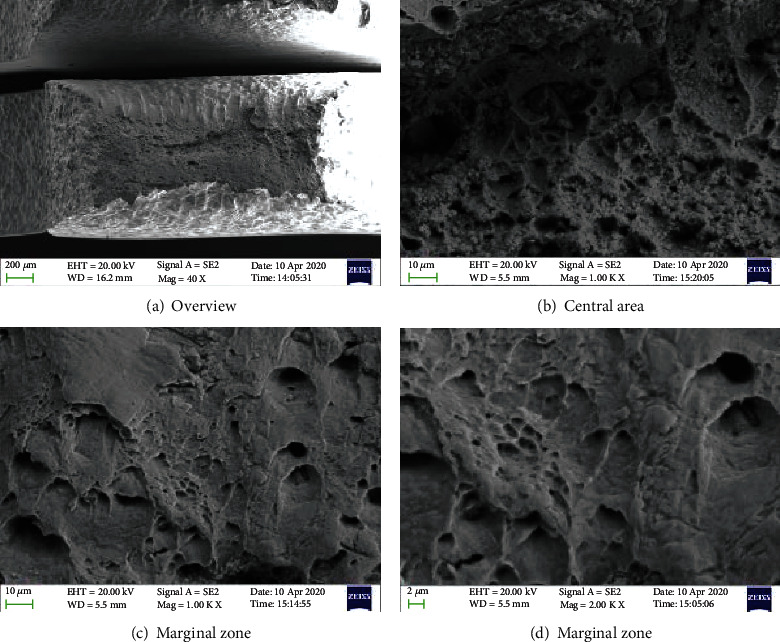
The fracture morphologies for TP 439 SS after SSRT test solution without FA.

**Figure 7:**
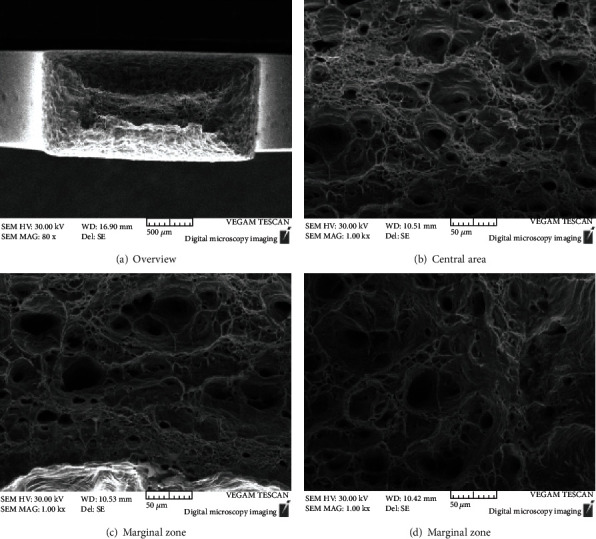
The fracture morphologies for TP 439 SS after SSRT in argon atmosphere.

Figures 5–7 show the morphology of the TP 439 SS in FA, non-FA, and argon, respectively. The fracture shows obvious necking, and it present a large number of dimples with holes, which do not make much difference. It shows that the use of FAs will not significantly affect the SCC behavior of TP 439 stainless steel, and the fracture is a ductile fracture, which is consistent with the results of its fracture mechanics performance curve.

#### 3.2.2. Fracture Morphology Analysis of 690 TT Alloy

**Figure 8:**
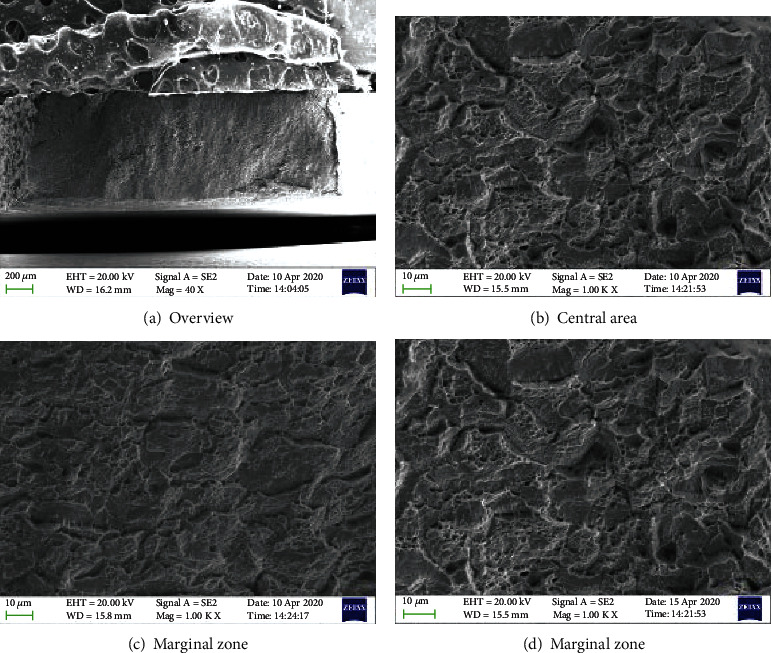
High temperature SSRT test fracture of 690 TT in the environment with FA.

**Figure 9:**
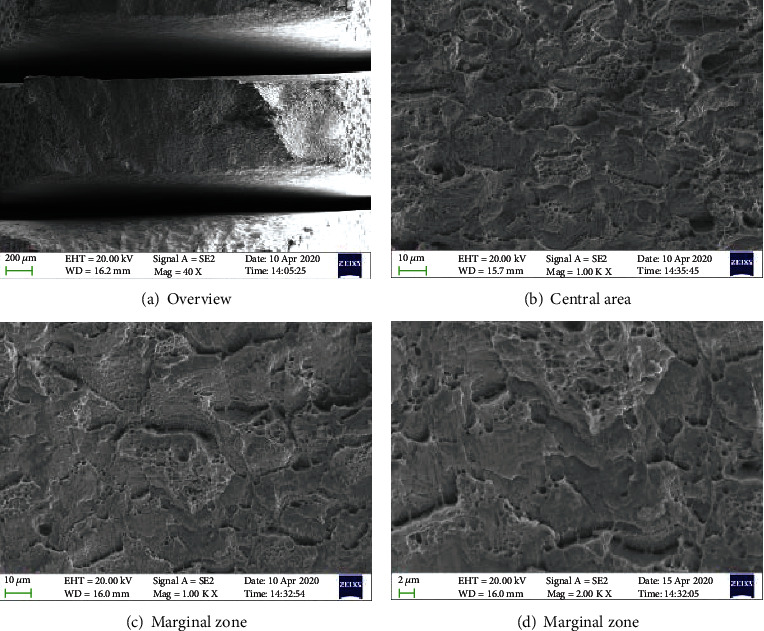
High temperature SSRT test fracture of 690 TT in the environment without FA.

**Figure 10:**
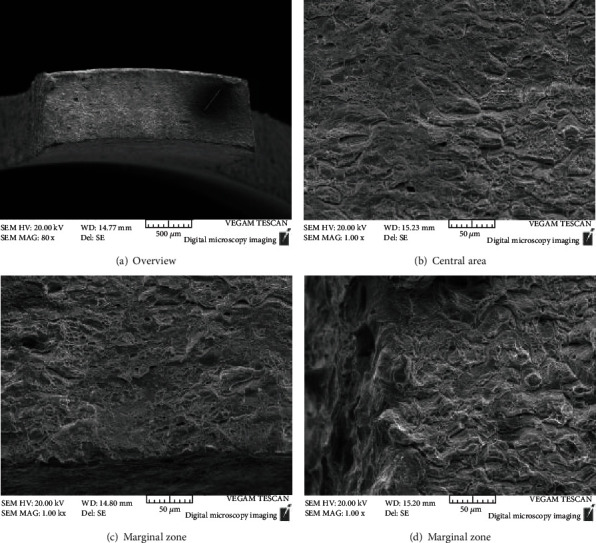
High temperature SSRT test fracture of 690 TT alloy under argon.

Figures 8–10 are the fracture morphologies of 690 TT alloy in FA, non-FA, and argon atmosphere, respectively. The fracture morphology of 690 TT alloy in the two environments is similar to that of the inert medium, and there is no obvious difference. Microscopically, to a certain extent, the fracture surface shows a mixed morphology of intergranular cracking and dimples. The reason for the intergranular cracking of 690 TT alloy is the TT treatment in the manufacturing process, which makes the 690 TT alloy grain boundary distribute a layer of continuous carbide M_23_C_6_, as shown in Figure 7, so it will show a certain degree of intergranularity under tensile stress. The cracking characteristics are not the appearance of intergranular stress corrosion cracking, which can be proved by the fracture in the argon atmosphere. Therefore, combined with the stress-strain curve of Figure 3, the 690 TT alloy does not exhibit stress corrosion characteristics in FA and non-FA environments.

## 4. Discussion

The test selected a commercial FA, as well as the heat exchange tube materials of the high-pressure heater and steam generator in the high-temperature operation area of a nuclear power plant: TP 439 stainless steel and 690 TT alloy. And the high-temperature slow strain rate test (SSRT) method was used to evaluate the effect of the FA on the stress corrosion cracking behavior of two metal materials. The test analyzes both mechanical properties and fracture morphology. The test results show that the fracture absorption energy of the sample is basically the same in FA and non-FA environments, so the FA used in the test will not significantly affect the SCC behavior of TP 439 stainless steel and 690 TT alloy under slow strain rate conditions within the test concentration range.

According to the existing practical application in the secondary circuit of nuclear power, the general dosage of FA is 1-2 mg/kg. This data is lower than the FA concentration used in the experiment. That is to say, from the practical application point of view, when the application concentration of FA is lower than 5 mg/kg, the test FA will not significantly affect the SCC behavior of TP 439 stainless steel and 690 TT alloy under slow strain rate conditions. Perhaps, this FA will have an impact on the SCC behavior of TP 439 stainless steel and 690 TT alloy, so the value of 5 mg/kg has not reached the threshold for this effect. This requires further research.

## 5. Conclusion

The test adopted the SSRT method. Under the environment of 5 mg/kg FA, SCC evaluation was carried out on two materials of TP 439 stainless steel and 690 TT alloy. The test results indicated that:

1. Under the condition of slow strain rate, the stress corrosion behavior of the two materials is relatively consistent in the two environments of FA and non-FA, and neither shows obvious stress corrosion sensitivity
2. Under the test conditions, the FA (<5 mg/kg) will not significantly affect the SCC behavior of TP 439 stainless steel and 690 TT alloy under the condition of slow strain rate
3. In actual application, the general dosage of FA is 1-2 mg/kg. This data is lower than the FA concentration used in the experiment. Therefore, there is no need to worry about the obvious impact on the SCC behavior of TP 439 stainless steel and 690 TT alloy

## Figures and Tables

**Table 1:** The chemical compositions of TP 439 SS and 690 TT alloy (wt%).

Element	Ni	Cr	Fe	C	Si	Mn	P	S	N	Al	Ti	Cu
690 TT	59.83	30.39	9.88	0.023	0.07	0.22	0.006	0.002	0.02	/	/	/
TP 439	0.247	17.84	81.06	0.014	0.179	0.262	0.025	0.0015	0.020	0.028	0.278	0.079

**Table 2:** The mechanical properties obtained from the SSRT test.

Material	Environment	Temperature *T*/°C	Maximum tensile strength *σ*_*b*_/MPa	Elongation rate *A*/%	Rate of reduction in area *Z*/%	Absorption area before fracture	Sensitivity index *I*_SCC_^∗^
TP 439	FA	260	388	21.9	48.6	91	6%
TP 439	Non-FA	260	388	11.4	53.6	89	8%
TP 439	Argon	260	412	27.1	89.1	97	/
690 TT	FA	284	570	45.4	70.6	245	9%
690 TT	Non-FA	284	568	39.0	68.8	242	11%
690 TT	Argon	284	661	45.4	53.8	272	/

^∗^The stress corrosion sensitivity index is calculated based on the fracture absorption energy.

**Table 3:** The SSRT test parameters.

Material	Environment	
TP 439 SS	FA	260°C, DO < 10 *μ*g/kg, 3.5 mg/kg NH_3_ pH 9.7, FA 5 mg/kg
	FA-free	260°C, DO < 10 *μ*g/kg, 3.5 mg/kg NH_3_ pH 9.7
	Argon	Inert gas environment

690 TT	FA	284°C, DO < 10 *μ*g/kg, 3.0 mg/kg NH_3_ pH 9.52, FA 5 mg/kg
	FA-free	284°C, DO < 10 *μ*g/kg, 3.0 mg/kg NH_3_ pH 9.52
	Argon	Inert gas environment

## Data Availability

The data used to support the findings of this study are available from the corresponding author upon request.
